# Left bundle branch potential predicts better electrical synchrony in bradycardia patients receiving left bundle branch pacing

**DOI:** 10.1186/s12872-022-02812-5

**Published:** 2022-08-19

**Authors:** Jingjuan Huang, Lina Guo, Weiwei Zhang, Ruogu Li, Ben He

**Affiliations:** 1 Department of Cardiology, Shanghai Chest Hospital, Shanghai Jiao Tong University, No. 241 W Huaihai Rd, Shanghai, 200030 China; 2 Department of Ultrasound, Shanghai Chest Hospital, Shanghai Jiao Tong University, No. 241 W Huaihai Rd, Shanghai, 200030 China

**Keywords:** Left bundle branch potential, Left bundle branch pacing, Bradycardia, Left ventricular electrical synchrony, Left ventricular mechanical synchrony

## Abstract

**Background:**

Left bundle branch pacing (LBBP) is a novel physiological pacing technology. We aim to explore the relation between LBB potential (LBB Po) and left ventricular (LV) electrical/mechanical synchrony in bradycardia patients without heart failure (HF) receiving LBBP.

**Methods:**

A total of 62 patients undergoing LBBP were categorized by LBB Po: the LBB Po positive (+) group and the LBB Po negative (−) group. The perioperative electrocardiographic and echocardiography parameters related to cardiac synchrony were analyzed.

**Results:**

There were 42 (67.74%) patients in the LBB Po (+) group and 20 patients in the LBB Po (−) group. Paced QRS duration (113.50 ± 17.65 ms vs. 123.40 ± 13.18 ms, P = 0.031) and stimulus left ventricular activation time (71.76 ± 3.53 ms vs. 74.45 ± 3.12 ms, P = 0.005) were shorter in the LBB Po (+) group than in the LBB Po (−) group. No significant differences in the LV mechanical synchrony (Ts-SD-12, 36.55 ± 19.76 vs. 39.95 ± 16.04, P = 0.505; PSD, 51.14 ± 17.69 vs. 45.65 ± 10.55, P = 0.205) between the two groups. There was not statistically difference in ventricular lead parameters measured intraoperative between the two groups. Compared with the LBB Po (−) group, the LBB Po (+) group showed a dramatically higher total procedure duration time (93.52 ± 9.18 min vs. 86.25 ± 10.54 min, p = 0.007) and fluoroscopy time for ventricle lead implantation (18.95 ± 3.43 min vs. 14.00 ± 3.16 min, p < 0.001).

**Conclusions:**

The appearance of LBB Po may suggest better electrical synchrony during LBBP, but similar in LV mechanical synchrony. However, the total operation duration and fluoroscopy time of ventricular lead implantation in the LBB Po (+) group were longer. Therefore, it may be unnecessary to deliberately recognize the LBB Po when it is difficult to detect LBB Po and meet the LBBP criterion.

## Background

Left bundle branch pacing (LBBP), defined as the capture of left bundle branch (LBB) via trans-ventricular septal approach, has been shown to be a novel physiological pacing modality with potential for application in both heart failure(HF) and conventional pacing patients [1–4]. During LBBP implantation, it is important to confirm that capture of the LBB has been achieved.

Left bundle branch potential (LBB Po), which is recognized as a high-frequency signal before the QRS onset and the interval between LBB Po and ventricular potential is usually 20–30 ms, was identified when the lead was implanted at left bundle trunk or proximal branch or left posterior fascicle or left anterior fascicle during intrinsic rhythm or LBBB correction by HBP [5]. The presence of LBB Po during LBBP implantation implies the lead is adjacent to the LBB, so the emergence of LBB Po is an important guide to perform LBBP [6]. However, according to previous studies, the proportion of LBB Po recorded varies greatly from 55.0 to 98.3% in different centers [2, 7–12]. Whether the absence of LBB Po, though the other LBBP criteria are met, will affect the cardiac electrical and mechanical synchronization is unclear and needs to be clarified.

The aim of our study was to explore the relationship between LBB Po and LV electrical/mechanical synchrony in bradycardia patients receiving LBBP. To this purpose, we evaluated electrocardiography (ECG) characteristics, two-dimensional Tissue Doppler imaging (2D-TDI) and two-dimensional speckle-tracking echocardiography (2D-STE) parameters in patients with and without LBB Po undergoing LBBP implantation.

## Methods

### Patient selection

The study prospectively enrolled pacemaker-indicated patients for symptomatic bradycardia without HF receiving LBBP at Shanghai Chest Hospital from March 2019 to August 2020 (ChiCTR1900020817) [13]. Patients were divided into LBB Po positive (+) group and LBB Po negative (−) group based on whether LBB Po was recorded. The institutional ethics committee approved the study protocol, and written informed consents were obtained from all subjects.

### LBBP implantation procedure and definition

LBBP was performed by screwing in a non-retractable active helix pacing lead from the right ventricular (RV) aspect into the interventricular septum as our previously described [1]. The C315His sheath (Medtronic Inc.) was moved 1–2 cm towards the RV apex direction in a RAO position along an imaginary line between the His region and RV apex. The pacing leads (Medtronic 3830, 69 cm), which were delivered through C315His sheath, were implanted in the LV septal sub endocardium of the LBB area.

Based on the electrical characteristics described by Huang and Chen et al. [6], LBBP was defined when the paced QRS morphology showed a “M” pattern in lead V1 and at least one of the following three conditions must also be present:

1. A transition from non-selective LBB capture to selective LBB or LV septal capture was distinguished while descending pacing output.
2. Abrupt shortening of stimulated left ventricular activation time (S-LVAT) > 10 ms was observed in lead V5 during the change of output.
3. If LBB Po could be recorded, the difference between S-LVAT and the interval from intrinsic LBB Po to R wave peak was ≤ 5 ms in V5. When LBB Po could not be recognized, the difference in V5 between the interval from the onset of paced QRS to R wave peak and the interval from intrinsic QRS onset to R wave peak was ≤ 5 ms.

We defined the time from first incision to last suture as the total procedure duration. The time from insertion of the 3830 lead and C315His sheath across the tricuspid valve to the appropriate lead final fixation site was called “fluoroscopy time for ventricle lead implantation”.

### Criteria of LBB Po

**Fig. 1:**
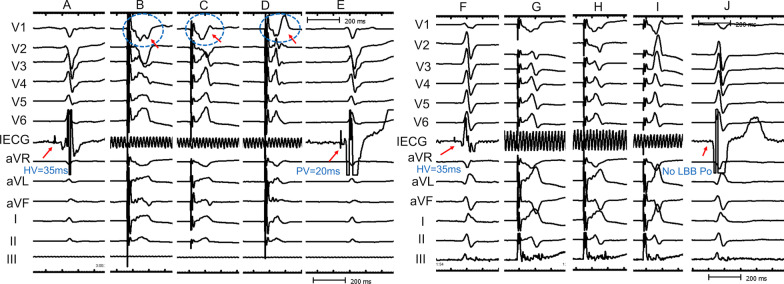
Implantation procedure and pacing parameters of LBBP. **A** and **F** His-bundle potential was identified at the RAO 30° and recording the fluoroscopic image of 3830 lead position as a reference. **B**–**D** and **G**–**I**, dynamic changes of “W” pattern in V1 lead during intermittent pacing of 3.5 V/0.5 ms when the 3830 lead was advanced inside of the septum. **E** LBB Po was measured at the final lead location. **J** there was no LBB Po at the final lead location. *LBBP* left bundle branch pacing; *LBB Po* left bundle branch potential; *RAO* right anterior oblique, *RBBB* right bundle branch block

During the operation, an intracardiac electrogram (EGM) was recorded from the 3830 pacing lead tip in a unipolar pacing mode to identify LBB Po. Intrinsic LBB Po has been considered to be a high-frequency signal about 20–30 ms before the surface QRS complex [5]. Amplitude of LBB Po were measured and the PV interval was also recorded from the LBB Po to the onset of the ventricular electrogram. To display LBB Po EGM, 200 mm/s was set as scanning speed on the GE CardioLab Electrophysiology recording system (unipolar recordings with bipolar pin box setup, 30 to 500 Hz) (Fig. 1).

### Programming of devices

The single-chamber pacemaker was used in patients with atrial fibrillation (AF) and programmed in VVI mode. The lower rate of the single-chamber pacemaker was routinely set at 75 beats per minute (bpm) and avoided fusion with intrinsic wave. For patients with sinus rhythm, the dual-chamber pacemaker was used and adjusted in DDD mode with short atrioventricular delay to ensure complete LBBP capture and avoid fusion with intrinsic conduction. To avoid septal anodal pacing, all ventricular electrodes were programmed in tip unipolar pacing manner with an output of 3.5 V/0.4 ms in the process of synchronization research. After the analysis of LV mechanical synchrony, all pacemakers were programmed according to the patients' specific condition to avoid the influence of Purkinje fiber conduction on RV activation.

### LV electrical synchrony evaluation and ECG study

Cardiac electrical synchrony between the ventricles was assessed using QRSd of 12-lead simultaneous body surface ECG recordings in lead V5 before and after the operation [14]. LV electrical synchrony was evaluated by S-LVAT [15]. Prolonged repolarization was recorded on ECG as lengthening of the QT interval, which was corrected for heart rate as corrected QT (QTc) interval using the Bazett formula [16]. Frontal plane QRS complex wave electrical axis needed to calculate the net amplitude in lead AVF and I by the following formula: QRS axis = 57.3 × ATAN (AVF/I). These ECG parameters were measured by two experienced and independent ECG specialists blinded to the study. Three continuous QRS complexes were analyzed, and the average value taken.

### Conventional echocardiographic assessment

Echocardiographic examinations were performed before LBBP operation using color Doppler ultrasonic diagnostic apparatus (Vivid E95 or Vivid E9, GE Healthcare, Horten, Norway). Echocardiographic measurements were performed by the same specialists according to the recommendations of American Society of Echocardiography [17]. All examinations were analyzed by two independent experienced investigators.

### Assessment of LV mechanical synchrony

Transthoracic echocardiography (TTE) was performed within 72 h after LBBP by the GE Vivid system. The echocardiographic data were stored and analyzed offline using an independent workstation (EchoPac for PC, GE Vingmed Ultrasound, Horten, Norway). LV mechanical synchrony was assessed by 2D-TSI and 2D-STE as described previously [18, 19].

In short, measurement of regional desynchrony was obtained from 2D-TDI images of the four-chamber apical view, and the time to peak myocardial systolic velocity (Ts) suggests the time to reach regional peak systolic tissue velocity. The time from QRS onset to Ts was recorded at the six basal and six midventricular level, including: (1) Ts-SD-12: standard deviation of Ts of the 12 LV segments; (2) Ts-12: maximal difference in Ts between any 2 of the 12 LV segments; (3) Ts-SD-6: standard deviation of Ts of the 6 basal LV segments; (4) Ts-6: maximal difference in Ts between any of the 6 basal LV segments.

Images from 2D-STE were obtained by an M5Sc transducer according to current consensus for all participants. An advanced quantitative analysis EchoPAC workstation was used for 2D-STE analyses. After 17 segments of the LV were successfully tracked, LV peak strain dispersion (PSD) was automatically analyzed and obtained.

### Statistical analysis

Continuous variables were expressed as mean ± standard deviation (SD) and classified variables were presented as percentages. Differences in mean values between two groups were compared by Student’s t-test for continuous variables. Classified variables were analyzed by the Fisher exact test, Chi-square test and Wilcoxon signed-rank test. Software SPSS 22.0 (SPSS Inc., Chicago, USA) was used to statistical analysis. A two-tailed P value < 0.05 was considered statistically significant.

## Results

### Patients’ characteristics

Between March 2019 to August 2020, there were a total of 62 pacemaker-indicated patients for symptomatic bradycardia without HF accepted LBBP therapy in our hospital. According to the defined criteria for LBB Po, there were 42 (67.74%) patients with LBB Po and 20 (32.26%) patients without LBB Po. Baseline characteristics of the two groups in pacemaker indication and echocardiography had no significant differences. The general information of the patients is shown in Table 1.

**Table 1:** Baseline patient characteristics

	LBB Po (+) N = 42	LBB Po (−) N = 20	P value
Male, N (%)	18 (42.85%)	8 (40.00%)	0.831
Age (years)	69.67 ± 11.32	67.90 ± 9.98	0.445
Hypertension, N (%)	25 (59.52%)	11 (55.00%)	0.738
CAD, N (%)	6 (14.29%)	4 (20.00%)	0.571
Diabetes, N (%)	9 (21.43%)	3 (15.00%)	0.552
Pacemaker indication			0.873
SSS	9 (21.43%)	4 (20.00%)	
AVB	23 (54.76%)	10 (50.00%)	
AF	10 (23.81%)	6 (30.00%)	
LAD (mm)	39.98 ± 5.29	39.45 ± 5.02	0.902
LVESD (mm)	30.74 ± 3.40	29.95 ± 3.71	0.399
LVEDD (mm)	48.86 ± 4.21	47.70 ± 3.45	0.578
LVEF (%)	64.29 ± 3.05	64.15 ± 3.20	0.900
BNP (pg/mL)	79.45 ± 52.02	98.40 ± 49.47	0.752

*AVB* atrioventricular block, *AF* atrial fibrillation, *BNP* B-type natriuretic peptide, *CAD* coronary artery disease, *LAD* left atrial diameter, *LBB Po* left bundle branch potential, *LVEDD* left ventricular end-diastolic diameter, *LVEF* left ventricular ejection fraction, *LVESD* left ventricular end-systolic diameter, *SSS* sinus node dysfunction. Other abbreviations are as in Fig. 1

### Procedure-related measurements

**Fig. 2:**
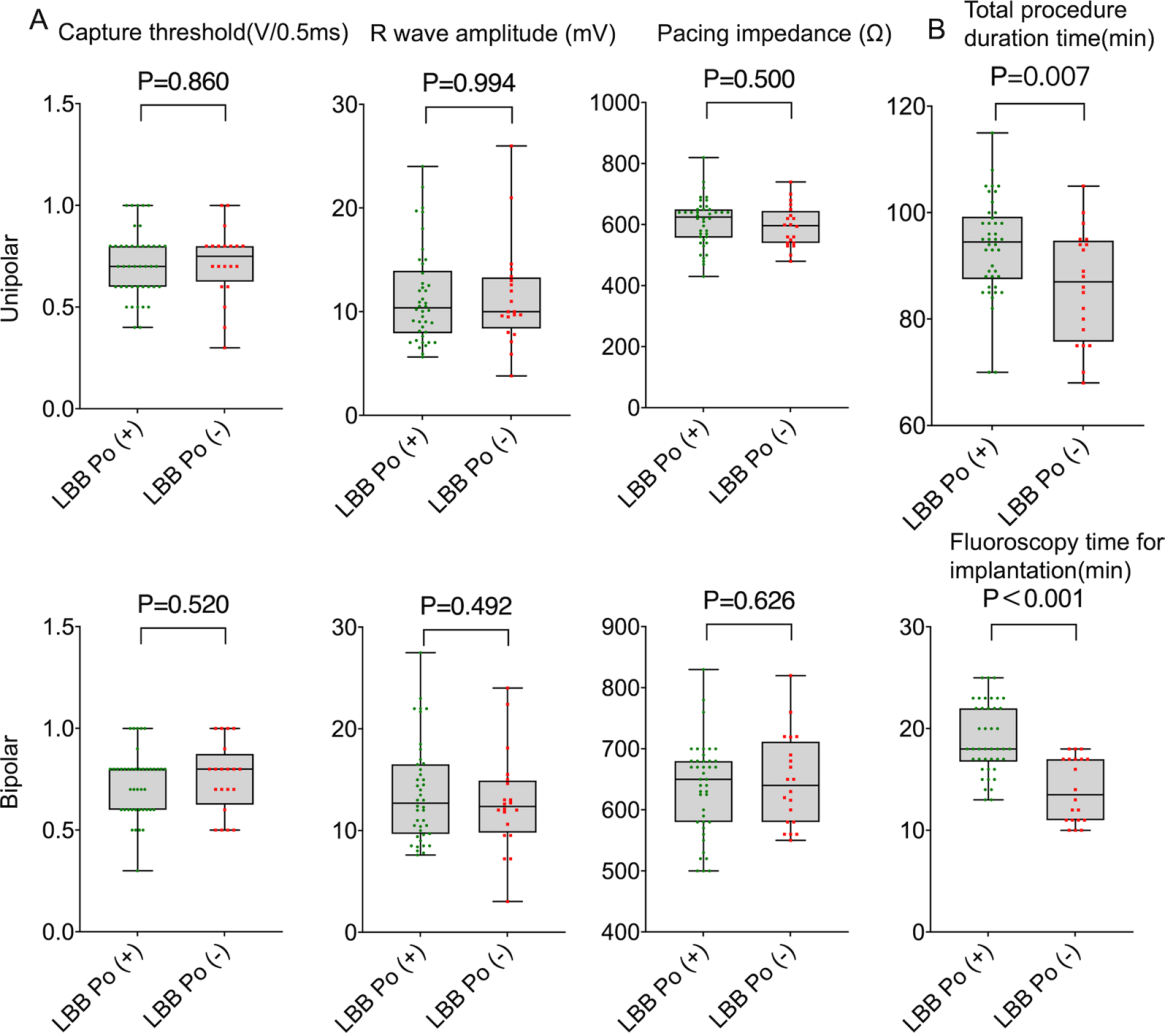
Procedural information in patients with LBBP.** A** There was no significant difference between LBB Po (+) and LBB Po (−) groups in ventricular lead capture threshold, R wave amplitude and pacing impedance.** B** Compared to the LBB Po (−) group, the LBBP (+) group showed a notably higher total procedure duration time and fluoroscopy time for ventricle lead implantation. Abbreviations are as in Fig. 1

LBB Po amplitude in LBB Po (+) cases were measured as 0.22 ± 0.05 mV, and mean PV interval was 25.40 ± 3.76 ms. Intraoperative measurement of the 3830 lead parameters demonstrated that there was no dramatically difference between LBB Po (+) and LBB Po (−) groups with regard to capture threshold (unipolar: 0.71 ± 0.16 V/0.5 ms vs. 0.72 ± 0.18 V/0.5 ms, P = 0.860; bipolar: 0.73 ± 0.16 V/0.5 ms vs. 0.76 ± 0.17 V/0.5 ms, P = 0.520), R wave amplitude (unipolar: 11.45 ± 4.62 mV vs. 11.44 ± 5.02 mV, P = 0.994; bipolar: 13.71 ± 4.90 mV vs. 12.79 ± 4.86 mV, P = 0.492) and pacing impedance (unipolar: 608.40 ± 78.17Ω vs. 594.50 ± 69.24Ω, P = 0.500; bipolar: 636.70 ± 76.17Ω vs. 646.80 ± 75.15Ω, P = 0.626) (Fig. 2A). Compared to the LBB Po (−) group, the LBBP (+) group displayed a markedly higher total procedure duration time (93.52 ± 9.18 min vs. 86.25 ± 10.54 min, p = 0.007) and fluoroscopy time for ventricle lead implantation (18.95 ± 3.43 min vs. 14.00 ± 3.16 min, p < 0.001) (Fig. 2B). The number of ventricle lead screw-in attempts between the LBBP (+) group and the LBB Po (−) group was significantly different (2.95 ± 0.85 vs. 2.30 ± 0.66, P = 0.004). No surgery-related complications were found in either group during the operation.

### Electrical synchrony evaluation

**Fig. 3:**
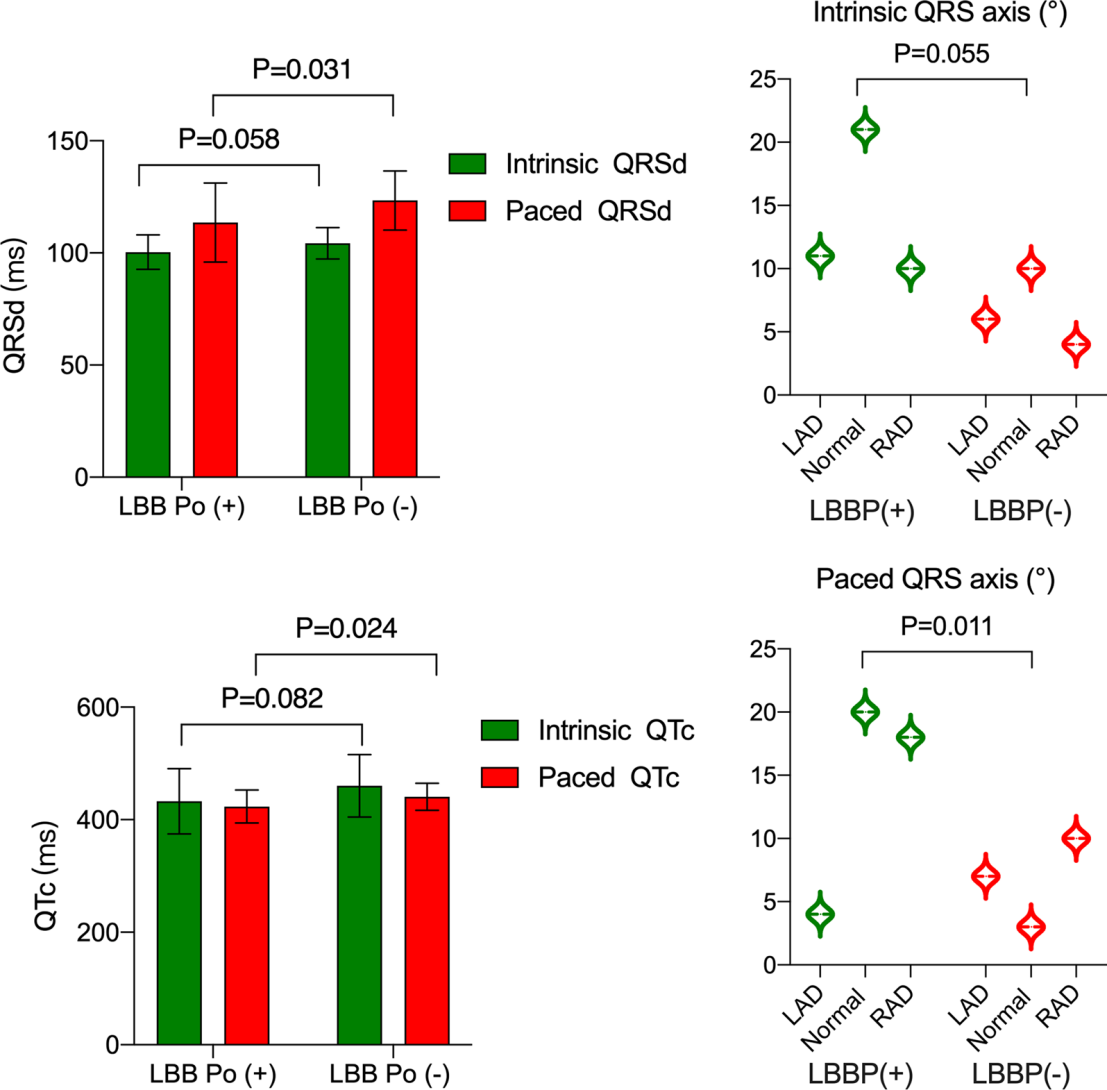
Comparison of the 12-lead ECG parameters pre-procedure and post-procedure. *LAD* left axis deviation, *QRSd* QRS durations, *QTc* corrected QT interval, *RAD* right axis deviation, *S-LVAT* stimulated left ventricular activation time; Other abbreviations are as in Fig. 1

**Fig. 4:**
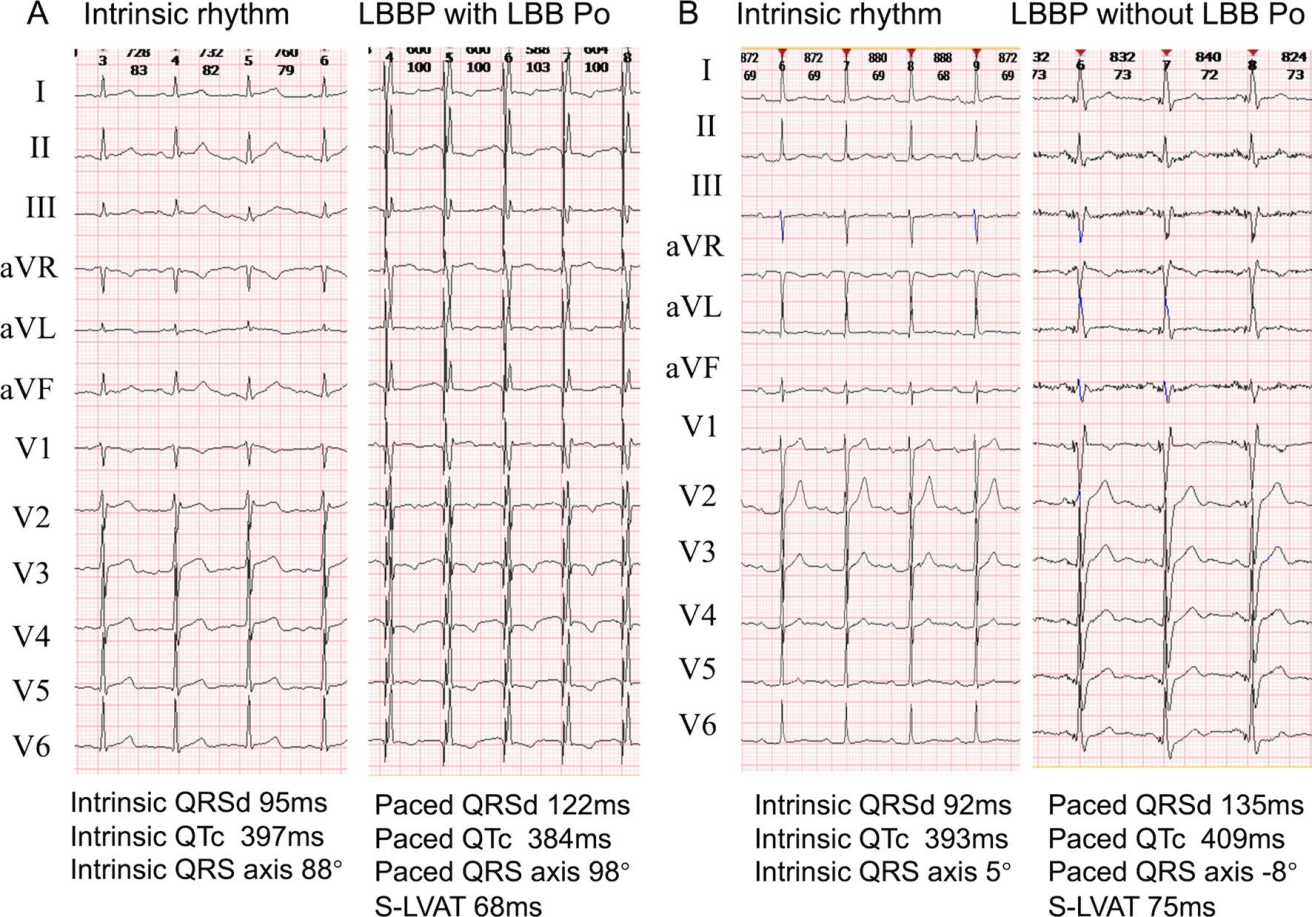
Examples of ECG of LBB Po (+) group and LBB Po (−) group. **A** QRSd of LBB Po (+) group was slightly wider than that in intrinsic rhythm. **B** QRSd of LBB Po (−) group was obviously wider than that in intrinsic status. Abbreviations are as in Fig. 3

The 12-lead surface ECG recordings pre-procedure showed that the intrinsic QRSd (100.40 ± 7.72 ms vs. 104.30 ± 7.05 ms, P = 0.058), QTc interval (432.80 ± 58.17 ms vs. 460.40 ± 55.47 ms, P = 0.082) and QRS axis (P = 0.055) did not significantly differ between LBB Po (+) and LBB Po (−) groups. In addition, patients in the LBB Po (+) group recorded a notably narrower mean paced QRSd than those in the LBB Po (−) group (113.50 ± 17.65 ms vs. 123.40 ± 13.18 ms, P = 0.031). Meanwhile, in the LBB Po (−) group, paced QTc interval (423.50 ± 29.36 ms vs. 440.90 ± 23.96 ms, P = 0.024) was prominently wider than those in the LBB Po (+) group. According to the paced QRS axis, patients were divided into three different types as follows: axis between − 30° and 90° (normal axis), axis between − 30° and − 90° (left axis deviation, LAD) and axis within 90° and 120° (right axis deviation, RAD). There was obviously significantly difference between LBB Po (+) group and LBB Po (−) group in paced QRS axis (P = 0.011). More patients belong to normal paced QRS axis in the LBB Po (+) group than in the LBB Po (−) group (Fig. 3). Mean S-LVAT of unipolar paced QRS complex (71.76 ± 3.53 ms vs. 74.45 ± 3.12 ms, P = 0.005) in LBB Po (+) group was significantly shorter than that of LBB Po (−) group. These typical examples showed the additional improvement in LV electrical synchrony when LBB Po was obtained (Fig. 4).

### LV Mechanical synchrony evaluation

**Fig. 5:**
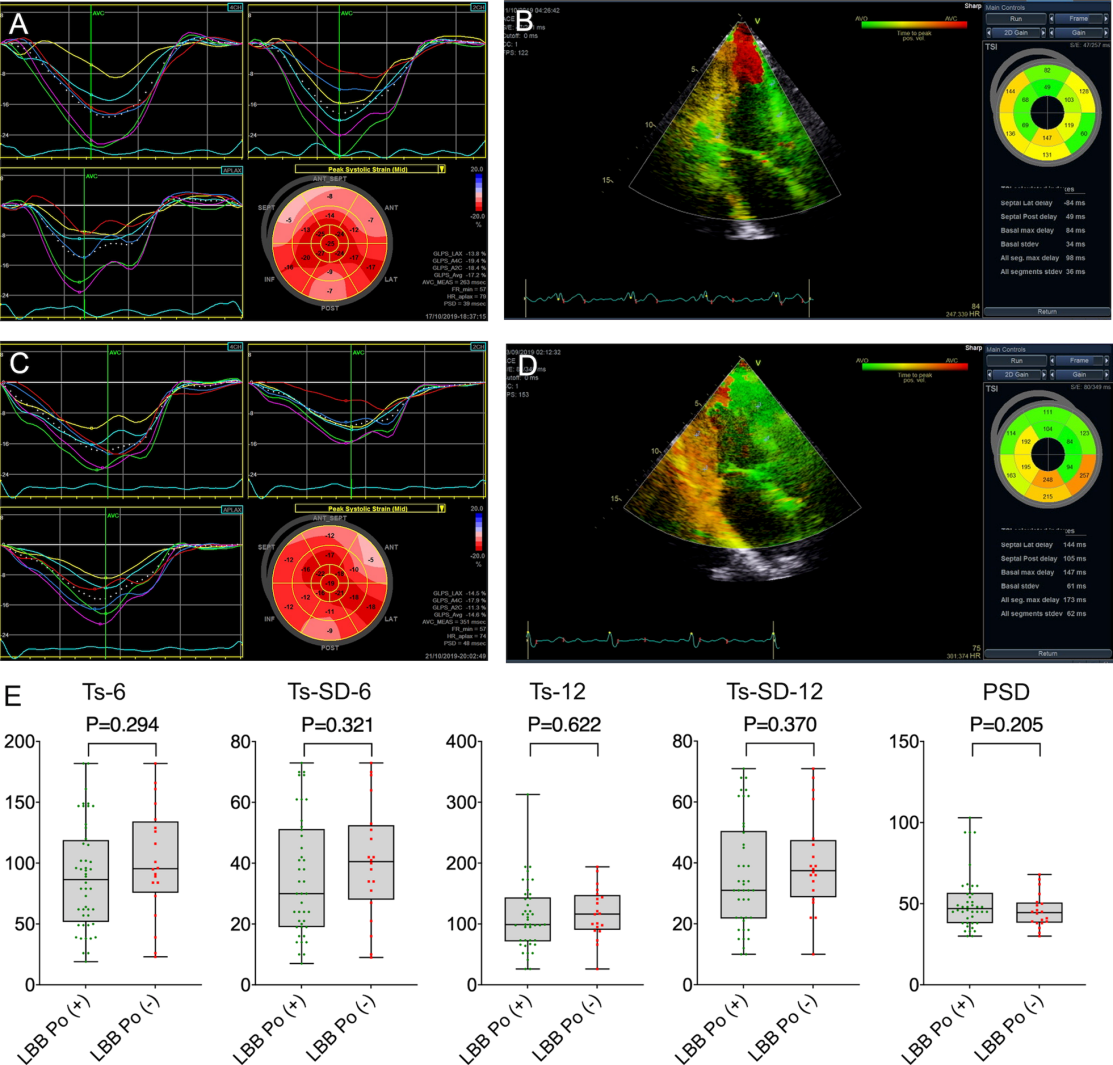
Comparison of LV mechanical synchronization parameters between LBB Po (+) and LBB Po (−) groups. **A**–**D** Examples of ECG of LBB Po (+) group and LBB Po (−) group. **E** Ts-6, Ts-SD-6, Ts-12, Ts-SD-12 and PSD were similar in the two groups. Ts, the time to peak myocardial systolic velocity; PSD, LV peak strain dispersion. Other abbreviations are as in Fig. 1

Patients in the two groups had similar LV mechanical synchrony. Our results showed that an obvious difference was not found in Ts-6 (88.83 ± 43.21 vs. 101.20 ± 45.13, P = 0.294), Ts-SD-6 (35.67 ± 18.07 vs. 40.65 ± 19.04, P = 0.370), Ts-12 (111.10 ± 55.12 vs. 118.10 ± 41.80, P = 0.622), Ts-SD-12 (36.55 ± 19.76 vs. 39.95 ± 16.04, P = 0.505) or PSD (51.14 ± 17.69 vs. 45.65 ± 10.55, P = 0.205) between the LBB Po (+) and LBB Po (−) group (Fig. 5).

### Follow-up

**Fig. 6:**
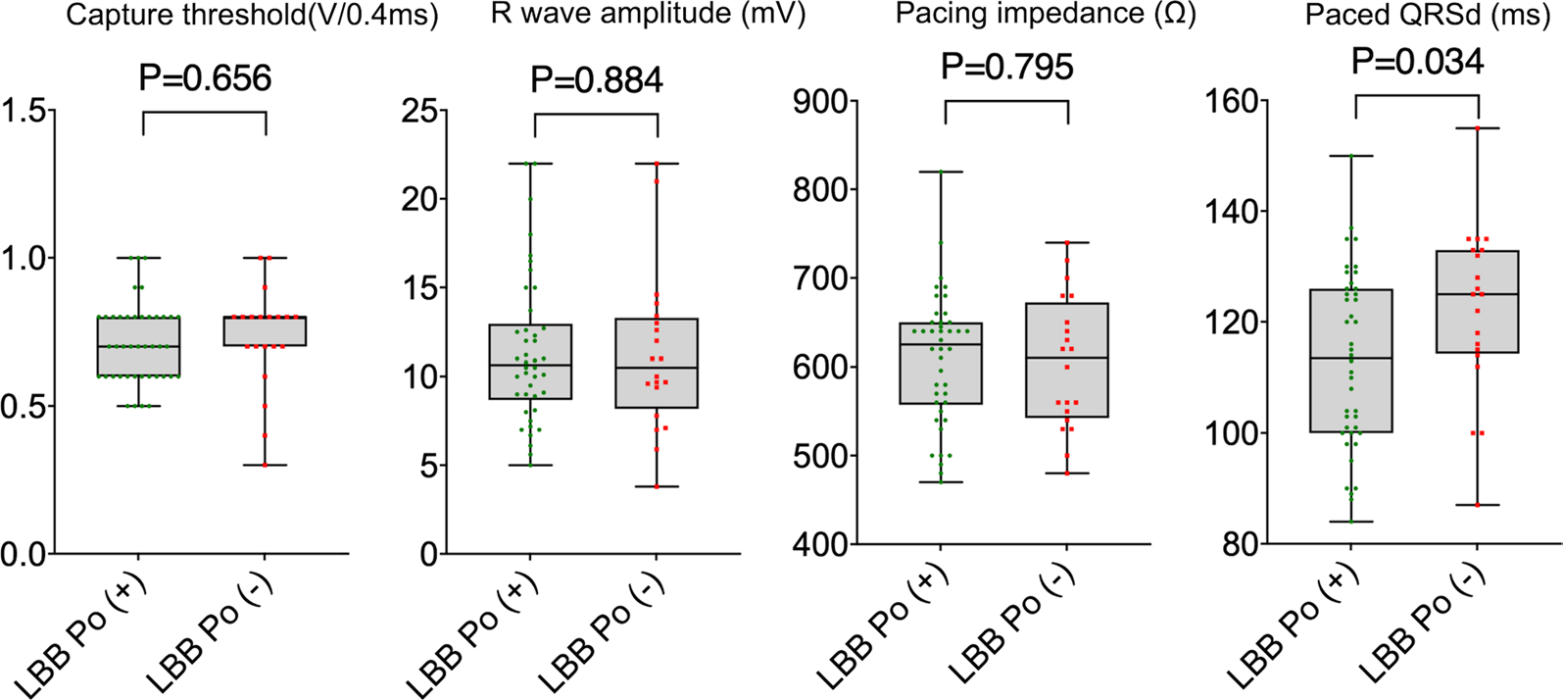
Follow-up at the 3-month. There was no significant difference in ventricular lead parameters but paced QRS in LBB Po (+) group was significantly shorter than that of LBB Po (−) group. Abbreviations are as in Fig. 1

ECG and ventricular lead parameters were evaluated in all patients at the 3-month follow-up. There was no significant difference in the R wave amplitude (11.40 ± 4.16 mV vs. 11.24 ± 4.50 mV, P = 0.884), capture threshold (0.71 ± 0.13 V/0.4 ms vs. 0.73 ± 0.18 V/0.4 ms, P = 0.656) or pacing impedance (609.80 ± 74.39Ω vs. 604.50 ± 75.15Ω, P = 0.795) between the LBB Po (+) and LBB Po (−) groups. Mean duration of unipolar paced QRSd (113.00 ± 15.86 ms vs. 122.30 ± 15.33 ms, P = 0.034) in LBB Po (+) group was significantly shorter than that of LBB Po (−) group. Two patients in the LBB Po (+) group were asymptomatic, in whom lead perforation was discovered incidentally on pacemaker interrogation. They cannot be paced at unipolar 10 V while the bipolar threshold increased abnormally. Echocardiogram showed that the 3830 pacing leads perforated through the interventricular septum (IVS), and the perforating leads were removed and replaced in the right ventricular apex by 5076 leads. No other serious complications such as lead dislocation, septal hematoma, coronary artery injury, LV thrombus, and stroke, or procedure-related death were observed during the follow-up (Fig. 6).

## Discussion

The major findings of our study can be summarized as follows: first, the presence of LBB Po meant better LV electrical synchronization in bradycardia patients receiving LBBP, although absolute differences are small. Secondly, it will take more time and rotation numbers to find LBB Po, but LBBP with and without LBB Po demonstrated similar LV mechanical synchrony. Those indicated that if other LBBP criteria are met, it may be unnecessary to take a long time to confirm LBB Po at implantation.

### Significance and influencing factors of LBB Po

His-Purkinje system pacing including HBP and LBBP is currently considered to be the optimal physiological pacing method with the pacing lead directly located into intrinsic conduction pathway to narrow QRSd and improve cardiac function. LBBP is considered an alternative approach for conduction system pacing, which can cross the blockage and ensure the left ventricular (LV) electrical synchronization with satisfactory pacing parameters [20]. Compared with HBP, LBBP has the advantages of better perception, lower acute and long-term thresholds, high R-wave amplitude, a relatively higher success rate, and wider range of patients [21, 22]. It is crucial to confirm LBB capture in performing LBBP.

In our research, the incidence of LBBP with LBB Po was about 67.74%, suggesting that LBBP patients accounted for about two-thirds of the total LBBP. The appearance of LBB Po suggested that the lead was close to the LBB and was a possible clue for the capture of the LBB. Therefore, it is generally believed that LBB Po is not a direct sign of LBB capture, but a good indication of LBB capture. Previous studies believe that in participants with an intrinsic non-LBBB rhythm, LBB Po always should be recognized when LBB capture is confirmed [12]. In subjects with complete AVB or intrinsic LBBB, LBB Po can be identified during an escape rhythm or ventricular premature contractions with right bundle branch block(RBBB) pattern [23]. As we know, it was inevitable that LBB Po could not be recorded in all patients, even in patients with the normal intrinsic conduction system. The recording rate of LBB Po may be affected by many factors, such as signal interference of far-field or near-field, velocity of conduction, electrode orientation, lead size, direction of wavefront, distance of the bundle branch, and the experience of operator [2]. In addition, the different clinical conditions of patients also make it difficult to identify LBB Po. LBB Po may be buried in ventricular EGM in patients with LBBB; nevertheless, these potentials can be recorded preceding the ventricular EGM by repairing of left bundle conduction via His corrective pacing [24]. Bigger current of injury or potential indicates that the electrode is related to a stable and lower threshold [11]. It may demand higher output to capture LBB in LBB Po negative patients, and LBB capture may be overestimated in these patients. In a patient with complete AVB and a baseline ventricular escape rhythm with RBBB pattern, HBP at a pacing output > 2 V/1 ms resulted in distal His bundle capture with RBBB correction [25]. This phenomenon suggested that the site of block was either intra-Hisian or intra-nodal. Thus, the LBBP lead tip was assumed to be implanted beyond the block area and distinguish the LBB Po during a stable RBBB escape rhythm. Nevertheless, possible explanations that absence of the LBB Po included: (1) though the LBBP lead had not reached the region of LBB, an output dependent QRS transition was misinterpreted as a QRS transition from NSLBBP to SLBBP. (2) There was a narrow escape rhythm beyond the blocked area, and the LBBP lead was located at the LBB distal to the blocked site. Consequently, the retrograde LBB Po was invisible because it might have been hidden in the ventricular EGM [25]. Of course, using electrophysiology catheters along the left side of the IVS would allow recording of anterograde LBB Po to different LBBP from LV septal pacing. Nevertheless, invasive electrophysiological measurement would increase the costs and operation procedure, so it was not suitable for routine use in daily clinical practice.

Previous studies have shown different LBBP characteristics due to the lack of unified criterion for evaluating LBB capture. LBB capture was usually identified by observing a transition of QRS complex from non-selective LBBP (NSLBBP) to selective LBBP (SLBBP) or LV septal pacing while descending output [6]. SLBBP needs to be differentiated by EGM, which is difficult to differentiate by a transition of QRS complex [6]. The other common parameters used to confirm proximal LBB capture include S-LVAT, SLBBP and paced RBBB pattern [21, 26–28].

### LBB Po and electrical synchrony

Electrical asynchrony is defined by an asynchronous electrical activation of the LV leading to a prolonged QRSd (> 120 ms) on the ECG. QRSd has been considered as an powerful indirect marker of electrical synchrony, and a wide QRS complex indicated greater ventricular dyssynchrony [29]. In the MOST trial, whether intrinsic or paced, a prolonged QRSd was related to an increased risk of HF [30]. Compared with normal QRSd, the prolongation of QRSd ≥ 120 ms was associated with more serious myocardial disease, worse prognosis, and higher all-cause mortality [29]. The results of PREDICT-HF study suggested that the paced QRSd negatively correlated with LVEF and had an adverse effect on long-term heart function [31]. In patients with an LVEF of 30% or less, prolonged QRSd implied increased mortality and sudden cardiac death. Furthermore, in patients with an LVEF of 30–40%, QRS prolongation was an independent predictor of increased mortality [32]. Therefore, according to current guidelines, QRSd is an important determinant for responses after cardiac resynchronization therapy [33]. Several studies have shown that LBBP can ensure a narrow QRSd, a stable threshold, and preserved electrical synchrony [22, 34, 35]. Our results suggested a significantly narrower QRSd in LBB Po (+) patients compared to LBB Po (−) patients, and the increasing QRSd indicated that the interventricular electrical synchronization becomes worse in the LBB Po (−) group. Therefore, LBBP with LBB Po can be converted to better heart function by restoring relatively normal interventricular electrical activation patterns. This is consistent with studies investigating RV pacing both in patients with HF and in patients with normal cardiac function [12]. Meanwhile, we should realize that QRSd may not be of great diagnostic value because it was affected by pacing site, myocardial capture, and distal conduction system diseases. Further study is needed to figure out electrophysiological characteristics of QRSd in LBBP with and without LBB Po.

### LBB Po and LV electrical synchrony

In the present study, the mean S-LVAT were shorter than 75 ms in all patients of LBBP. LBBP patients without LBB Po had longer S-LVAT than those with LBB Po, although the difference in the absolute value is small. In Hou’s study, LBBP patients with recorded LBB Po almost had a shorter S-LVAT and a better LV electrical synchrony compared with those without LBB Po. But there are still 66.7% of patients with long S-LVAT had worse LV mechanical synchrony than those with short S-LVAT in LBB Po negative cases [8]. As we known, the conduction velocity of His-Purkinje system is faster than that of ventricular myocardium. Once the LBB is active, the S-LVAT should maintain short and constant regardless of the output. We inferred that LBBP without LBB Po only captured the small branches of LBB, or only obtained the LV deep septal pacing, or even only activated LV local endocardium. Possible explanations come from previous studies, suggesting that electrical activation of the working myocardium starts at the endocardium of LV septum and captures superficial endocardial fibers, not necessarily Purkinje fibers, leading to relative physiological ventricular activation [36, 37]. These results showed that short S-LVAT can be used as a useful reference index to guide LBBP implantation when LBB Po negative.

### LBB Po and LV mechanical synchronization

As shown in our study, LBBP patients with LBB Po had obvious advantages over LBBP without LBB Po in S-LVAT and electrical synchronization, but there was no significant difference in LV mechanical synchronization. This result was in agreement with Wang's finding [34]. Our study was short-term efficacy evaluation of mechanical synchronization. We adopted echocardiography to estimate LV mechanical synchrony by 2D-TDI and 2D-STE. Ts-SD-12 is the most widely used parameter to evaluate LV desynchrony, which more than 31.4 ms was defined as intraventricular asynchrony [38]. PSD is used to assess the early LV systolic dysfunction by combining the synchronization and coordination of cardiac mechanical movement. LV mechanical synchronization parameters (Ts-12, Ts-SD-12, Ts-6, Ts-SD-6, PSD) were similar in LBB Po (+) and LBB Po (−) groups. And LBB Po (−) group had a slightly longer Ts-SD-12 and worse LV mechanical synchronization than LBB Po (+) group, but a statistically significant difference was not discovered. This may be owing to the relatively small study samples and short-term follow-up, and future studies with a larger sample size and long-term follow-up are needed to confirm this effect. Of course, the decision of pacing strategy needs to consider the percentage of ventricular pacing and basic heart function. Hou et al. pointed out that when a high-burden of RV pacing was expected, with a high risk of pacing-induced cardiomyopathy or cardiac synchronization therapy was needed, LBBP with LBB Po should be considered instead of LBBP without LBB Po [8]. Further long-term follow up and large-scale randomized studies are needed.

### Other ECG parameters and follow-up

The frontal QRS axis represents the main direction of ventricular electrical depolarization on frontal plane. In pacemaker-dependent patients, normal paced QRS axis could provide relatively physiological ventricular activation associating with the preservation of LV function [39]. Recent paper has also shown that the abnormal paced QRS axis may cause damage of cardiac function in RVP [39]. In our study, there were more patients with normal paced QRS axis in the LBB Po (+) group than in the LBB Po (−) group. The pacing area of LBBP is usually located in the left bundle trunk or proximal branch, left posterior fascicle, and left anterior fascicle. Left axis deviation of the paced QRS axis may be caused by capturing the left posterior fascicular branch.

Prolongation of QTc interval was associated with an increased risk of severe arrhythmia and cardiac death [40]. Meanwhile, the paced QTc interval appears to be a useful marker in predicting poor prognosis. In patients with preserved LV systolic function, prolonged paced QTc interval related to new LV systolic dysfunction and cardiac death after permanent pacemaker implantation. The rate of cardiac death increased significantly, especially in patients who showed prolonged paced QTc along with new onset LV systolic dysfunction [41]. The present study showed that paced QTc interval was more shortened in LBB Po (+) group than that in LBB Po (−) group. The relationship between paced QTc interval and clinical prognosis is unclear in patients undergoing LBBP implantation. Therefore, long-term follow-up might be required for patients who represented longer paced QTc interval after LBBP, especially in LBBP without LBB Po.

We followed up the lead parameters and only two patients in LBB Po (+) group suffered from lead perforation, while all other LBBP patients had stable sensing amplitude, capture threshold and lead impedance. There are no other serious complications reported at three-months follow-up in the two groups. However, the total operation duration and fluoroscopy time of ventricular lead implantation in the LBB Po (+) group were longer. We think that the excessive pursuit of the short S-LVAT and the capture of the LBB may lead to excessive lead rotation and increase the risk of lead perforation. Therefore, it may be unnecessary to deliberately recognize the LBB Po when it is difficult to detect LBB Po and meet the LBBP criterion.

### Study Limitations

This study is a small sample size, single-center and short-term study, possibly causing an underestimation of the actual effects. Noise signals may affect the acquisition of intracavity electrical signal, thus affecting the judgment of LBB Po. The parameters used in this paper to describe the electrical and mechanical synchronization are not comprehensive and need to be further improved. There may be inevitable errors in the measurement parameters of ECG and echocardiography. Whether the long-term clinical effects of LBBP mechanical synchronization change after echocardiography in a short time needs to be further investigated. More detailed analysis may be based on parameter differences between spontaneous and paced status and relation between them. For future analysis may be important the longer follow up and the analysis of prolonged QTc interval. The validity of the conclusion needs to be further demonstrated by using more accurate techniques and expanding the sample size in the future.

## Conclusions

LBB potential is commonly observed during LBBP and can be associated with a better LV electrical synchronicity in bradycardia patients with normal cardiac function. However, LBBP with and without LBB Po resulted in similar cardiac mechanical synchrony, indicating that it may be unnecessary to deliberately recognize the LBB Po when it is difficult to detect LBB Po and meet the LBBP criterion.

## Data Availability

Data are available from the corresponding author upon reasonable request due to privacy or other restrictions.

## Abbreviations

AF: Atrial fibrillation
AVB: Atrioventricular block
BNP: B-type natriuretic peptide
CAD: Coronary artery disease
IVS: Interventricular septum
LAD: Left atrial diameter
LBBB: Left bundle branch block
LBB Po: Left bundle branch potential
LV: Left ventricle
LVEDD: Left ventricular end-diastolic diameter
LVEF: Left ventricular ejection fraction
LVESD: Left ventricular end-systolic diameter
PSD: LV peak strain dispersion
QRSd: QRS durations
QTc: Corrected QT interval
RAD: Right axis deviation
RAO: Right anterior oblique
RBBB: Right bundle branch block
S-LVAT: Stimulated left ventricular activation time
SSS: Sinus node dysfunction
Ts: The time to peak myocardial systolic velocity
